# Multiples kératoses séborrhéiques de siège inhabituel

**DOI:** 10.11604/pamj.2014.18.267.4994

**Published:** 2014-08-01

**Authors:** Ahlam Abdou, Badreddine Hassam

**Affiliations:** 1 Service de Dermatologie, CHU Ibn Sina, Université Med V, Souissi, Rabat, Maroc

**Keywords:** Kératoses séborrhéiques, verrue, biopsie cutanée, seborrheic keratoses, wart, skin biopsy

## Image en medicine

La kératose séborrhéique (KS), anciennement appelée verrue séborrhéique ou verrue séborrhéique papillaire hyperkératosique, est une tumeur cutanée bénigne, qui survient essentiellement chez le sujet âgé au-delà de 50 ans. Elle est fréquente sur le tronc, le visage et rare dans la région génitale. Elles forment des lésions en relief, bien limitées, comme posées sur la peau, d'aspect squamo-kératosique, de coloration brun-gris à noire. Elles se détachent facilement à l'aide d'une curette. Elles sont souvent multiples et leur taille peut atteindre plusieurs centimètres. Dans cette localisation le principal diagnostic différentiel c'est les verrues génitales à human papilloma virus. Leur étiopathogénie est inconnue. Elles pourraient survenir après une dermatose inflammatoire ou sur un site de frottement répété. Nous rapportons le cas d'un patient âgé de 40 ans, sans antécédents pathologiques particuliers, consultait pour des lésions pubiennes pigmentées évoluant depuis 5 ans. L'examen clinique retrouvait de nombreuses lésions noirâtres à surface kératosique cérébriformes évoquant des kératoses séborrhéiques siégeant au niveau des organes génitaux externes et au niveau du pubis. Les diagnostics évoqués étaient des KS géantes et des condylomes. Une biopsie cutanée d'une lésion papuleuse noirâtre montrait une acanthose épidermique associée à une orthokératose s'invaginant par endroit dans le corps muqueux en réalisant des puits caractéristiques. Un traitement par électrocoagulation-curetage a été réalisé.

**Figure 1:**
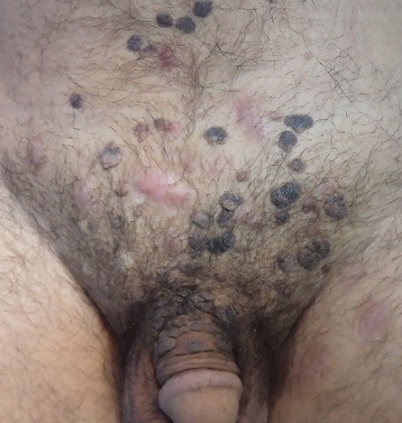
Multiple kératoses séborrhéiques de siège scrotale

